# The curious case of the Cat Rescue: can picture narrative description inform visuospatial processing in aphasia?

**DOI:** 10.3389/fnhum.2025.1574453

**Published:** 2025-05-22

**Authors:** Sarah Grace Dalton, Javad Anjum, Davida Fromm, Brian MacWhinney

**Affiliations:** ^1^Department of Communication Sciences and Special Education, University of Georgia, Athens, GA, United States; ^2^Department of Psychology, Carnegie Mellon University, Pittsburgh, PA, United States

**Keywords:** aphasia, discourse, visuospatial processing, core lexicon, aphasia severity

## Abstract

**Background:**

Examining discourse production during picture description performance holds great promise for understanding the nature of and the interconnectedness between visuospatial processing and language production in aphasia – a language disorder following acquired brain damage. There is a paucity of studies concurrently investigating the two processes in discourse production tasks, despite their potential clinical utility in aphasia rehabilitation. In the current study, we compared the core lexicon (CoreLex) word production performance of PWA and matched healthy control participants (HCP) along the dimensions of typicality of words (e.g., the words most frequently used by a normative sample of healthy controls), CoreLex word production timing, as well as the indirect visuospatial measures of order and spatial location of CoreLex word productions across the four quadrants of the Cat Rescue picture in a story telling task.

**Methods:**

A total of 319 transcripts from HCP and 400 transcripts from PWA, all of whom completed the picture description task of the Cat Rescue were drawn from the AphasiaBank database—the largest repository of aphasic discourse samples in the world. For each transcript, CoreLex scores and timing data of word production – including the elapsed time to produce each core lexicon content item and the first core lexicon content word in each quadrant—were indexed across the four quadrants of the Cat Rescue picture using the CLAN (Computerized Language ANalysis) program.

**Results:**

CoreLex analysis revealed that PWA were significantly slower compared to HCP in producing the first CoreLex word for each quadrant of the picture. PWA also demonstrated delayed CoreLex word production times across all the four quadrants, as well as lower rates of certain CoreLex word production compared to HCP. Aphasia severity was inversely related to the latency and accuracy of CoreLex production.

**Conclusion:**

Study findings offer preliminary evidence for the clinical utility of integrating concurrent visuospatial processing and language production tasks as part of discourse assessment in PWA. Future implications for further expanding and refining discourse-based visuospatial processing assessment tools in aphasia rehabilitation are discussed.

## 1 Introduction

Visuospatial and language processing are two distinct yet interconnected domains that are essential for human communication. The typical pattern of functional lateralization of visuospatial processing in the right hemisphere and language processing in the left hemisphere has led researchers to focus predominantly on visuospatial processing as a function of the right hemisphere and language processing as a function of the left hemisphere (Vingerhoets, 2019; Quin-Conroy et al., 2024). In keeping with this view of functional lateralization, most empirical studies in clinical populations have been limited to exploring the connection between visuospatial abilities and language in individuals with primarily right hemisphere damage (e.g., Bartels-Tobin and Hinckley, 2005; Minga et al., 2022) or individuals with bilateral damage, whether due to traumatic brain injuries or dementia (e.g., Foxe et al., 2013; Watson et al., 2018). Few studies have explored the interconnectedness of visuospatial processing and left hemisphere language function, despite compelling evidence of their crucial involvement in communication functions in people with and without brain injuries, as well as those with hearing impairments (Marini, 2012; Rudner et al., 2016; Diedrichs et al., 2022).

Contrary to the historical view of hemispheric independence, people with aphasia (PWA) often present with co-occurring deficits in nonlinguistic cognitive domains. Helm-Estabrooks (2002) provided a framework for categorizing these deficits under the domains of memory, attention, executive functions, and visuospatial skills, emphasizing their importance in aphasia assessment, treatment, and long-term rehabilitation. Since then, several studies have investigated the intricate relationships between language processing and nonlinguistic cognitive abilities of memory (Ivanova et al., 2017; Silkes et al., 2021; Wright et al., 2007), attention (Hula and McNeil, 2008; Lee, 2020; Murray, 2012), and executive functions (Frankel et al., 2007; Olsson et al., 2019; Purdy, 2002) in aphasia. There is a need to understand potential connections underlying language and visuospatial processing in aphasia, for four reasons. First, there is evidence to suggest a direct correlation between the extent of visuospatial deficits and the severity of language impairments. These deficits affect both comprehension and production domains and have been observed in individuals with damage to either the left or right hemisphere of the brain (Hwang et al., 2022; Pyun et al., 2009; Trojano et al., 2004; Yao et al., 2020). Second, the evidence shows that despite severe cognitive and language impairments following stroke, visuospatial cognitive functions may be spared when compared to some of the other nonlinguistic cognitive functions (Marinelli et al., 2017). Third, visuospatial learning may be a significant predictor of aphasia treatment outcomes (e.g., improving naming and comprehension) as well as patient response to treatment (Diedrichs et al., 2022; Dignam et al., 2017; Gilmore et al., 2019; Seniów et al., 2009). Fourth, there is a scarcity of empirical studies investigating concurrent language production and visuospatial processing behavior in PWA. Much of the existing evidence in this line of research comes from correlational studies exploring performance of PWA on standardized cognitive-linguistic assessments and using these findings to develop predictions on language outcomes. While some relationships between visuospatial and language processing skills may arise incidentally due to the common use of visual stimuli in assessment and intervention practices in language rehabilitation, there is a need to intentionally examine their simultaneous involvement within ecologically valid, discourse level tasks. Addressing this issue is also timely, as most studies in aphasia have been restricted to correlational investigations of language comprehension and visuospatial behavior (Heuer et al., 2017; Smith et al., 2018).

In people without brain injuries, research exploring the connections between language processing and visuospatial skills is mostly rooted in eye-tracking studies. There is evidence to show that while low-level features such as luminance, color, orientation, and contrast of scenes can potentially influence automatic eye movements of viewers to some extent via bottom-up processes (perceptual features), the majority of natural eye movement behavior is guided by top-down processes. Viewers’ attention to different parts or visual objects within a scene are influenced by cognitive factors, such as task goals, short-term memory, and prior knowledge of the scene components (Cronin et al., 2020). Thus, naturalistic visual scene perception contexts predominantly draw viewers’ attention to more information-rich and meaningful parts of the scene, allowing us to study the complex interrelationships between language processing and visuospatial behavior in both people with and without aphasia (Altmann and Kamide, 2009; Henderson and Hayes, 2018; Heuer et al., 2017; Smith et al., 2018). While most eye-tracking studies have investigated connections between language comprehension and visual behavior related to scene perception (also called visual world paradigm), there is a lacuna of empirical investigations studying language production and visuospatial abilities, especially in PWA. Seminal eye-tracking studies investigating language production and visual behavior in people without aphasia have suggested that eye movements are closely coordinated with speech production (Tanenhaus, 2007). Viewers typically tend to fixate on a single object for a duration of about 1,000 ms prior to naming the object (Rossion and Pourtois, 2004) and about 800 ms – 1,000 ms before beginning to describe a simple event (e.g., lightning striking a building) in an active or passive sentence (Griffin and Bock, 2000). These unique patterns of *eye-voice delay* represent word preparation before picture description (Tanenhaus, 2007). However, it is difficult to draw conclusions regarding language performance and visuospatial skills based on tasks depicting isolated contexts, (e.g., single word or simple events). A discourse task such as picture description, which reflects everyday communication demands and their associated cognitive loads, offers a promising window into understanding the multifaceted connections between language production and visuospatial behavior in aphasia (Dalton et al., 2020b; Dalton et al., 2024). This relationship between visuospatial abilities and discourse also lends itself well to investigating either process using the other as a tool, as in a picture description task, wherein a participant is engaged in producing a discourse sample that corresponds to a visual stimulus. Varied examples of such paradigms are available in the existing literature, across several neurogenic cognitive and communicative disorders. They include aphasia (Naranjo et al., 2023), mild cognitive impairment (MCI) (Kim et al., 2019), Alzheimer’s Dementia (AD) (Fromm et al., 2024; Kim and Lee, 2023; Kintz et al., 2024), primary progressive aphasia (PPA) (Seixas-Lima et al., 2022), right hemisphere damage (RHD) (Minga et al., 2022), and mild traumatic brain injury (mTBI) (Myers et al., 2022). However, the current scarcity of discourse-level language tasks investigating cognition in aphasia can be attributed to four factors. First, there is a paucity of standardized procedures for collection, transcription, and analysis of discourse data. Further, a lack of normative data for discourse measures poses challenges for replication of studies, comparison across studies, and interpretation of results for PWA (Bryant et al., 2017; Dietz and Boyle, 2017; Dipper et al., 2021; Linnik et al., 2016; Stark et al., 2021a,b). Second, barriers to clinical practice limit the integration of discourse assessment and treatment procedures in PWA. They include time constraints (Bryant et al., 2017), inadequate access to discourse knowledge, skills, tools, training protocols, and expertise to process large numbers of discourse samples (Cruice et al., 2020; Cruice et al., 2021). Third, historically aphasia is conceptualized and studied predominantly as a language disorder, although some investigations exploring the complex interrelationships between linguistic deficits and cognitive functions are available (e.g., Helm-Estabrooks, 2002; Marinelli et al., 2017). Finally, there is limited clinical or research incentive for concurrently studying aphasia discourse and cognition, which can be further attributed to a current scarcity in empirical studies. Most of the evidence in this line of research originates from RHD and dementia literature, given that the nature of symptoms often subsumes concomitant cognitive and linguistic abilities.

In RHD, it is reported that individuals with visuospatial deficits often experience a wide array of challenges when performing a discourse task, such as picture description. These include problems related to sequencing events, maintaining coherence, and focusing on the key details of the visual stimuli (Dalton et al., 2024; Marini, 2012; Rivers and Love, 1980). Further, two studies have reported novel correlations between visuospatial processing and narrative language abilities, which further highlights the feasibility and validity of investigations that concurrently study visuospatial and discourse production abilities in people with brain injuries (Minga et al., 2022; Bartels-Tobin and Hinckley, 2005). Minga et al. (2022) found that visuospatial abilities may contribute to question-asking behaviors during conversation, although only modestly. Bartels-Tobin and Hinckley (2005) reported that both the clock-drawing task and visuospatial processing domain score on the Cognitive Linguist Quick Test (CLQT) were related to how well individuals with RHD produced the “gist” or main concepts of a narrative discourse task (the Cinderella storytelling). Further, recent investigations in individuals with Alzheimer’s Dementia (AD) have shown that visuospatial processing features during the production of core lexicon words when performing discourse tasks hold potential in improving the diagnosis and classification of AD (Ambadi et al., 2021; Fromm et al., 2024;; Heidarzadeh and Ratté, 2023; Zuo et al., 2024).

While conversation is the gold standard for discourse analysis, the lack of visual support limits its use in the current context. Picture-supported discourse tasks may provide enhanced insights into how visuospatial processing relates to communication challenges, including poorer sequencing and organization of language. Prior research has suggested that storytelling tasks using pictures elicit lexically diverse language production. Visual support features of the pictures evoke concrete and high imageability words contributing to the production of varied words (Fergadiotis, 2011; Fergadiotis and Wright, 2011). Additionally, they provide a context for the narrative, including setting, action, sequence of events, and actors—all of which are also known to collectively evoke an emotional response from the narrator as the story unfolds (Kim and Wright, 2020), further enhancing the lexical diversity of the words produced.

Core lexicon (CoreLex) analysis measures the typicality of words used in structured monologic discourse samples and entails comparison of a person’s lexical choice in a structured discourse task with a checklist that includes core words most commonly produced by people without brain injuries for the same task (Dalton et al., 2022; Dalton et al., 2024). CoreLex checklists have been developed for multiple structured discourse tasks (e.g., picture scene narratives, picture sequence narratives, story retells, procedures) using large samples of speakers without brain damage (Dalton et al., 2020b; Dalton et al., 2024). These checklists include both content (e.g., nouns, verbs, adverbs, adjectives) and function (determiners, prepositions, pronouns, etc.) words. It is important to note that CoreLex scores do not include credit for production of synonyms or circumlocutions for CoreLex items. Therefore, scores may not accurately reflect the overall informativeness of a discourse sample. It is possible for an individual to produce synonyms that communicate the gist of a story without producing core lexicon items. Rather, core lexicon analysis allows us to determine whether patients can retrieve the words most commonly used by healthy controls to produce a discourse. The success of lexical retrieval (measured by CoreLex) in picture description tasks can be influenced by the visuospatial demands and the individual’s ability to process the visual elements during language production. While CoreLex analysis focuses on the lexicon used to describe these concepts, the initial identification of what is important to describe relies on visuospatial processing, as difficulties in visual attention may influence the selection of core lexical items.

Existing evidence points to several advantages of using CoreLex analysis in clinical populations, especially in PWA. First, CoreLex analysis is efficient in that it is easy to administer and score without the need for transcription. Thus, it is suitable for use by busy clinicians (Dalton et al., 2020b; Fromm et al., 2024; Kim and Wright, 2020). Second, CoreLex analysis measures offer sensitive and reliable indices for discerning discourse differences between healthy speakers and PWA (Kim et al., 2021). Third, CoreLex analysis provides sensitive indices that measure treatment-induced changes (Kim et al., 2021), as well as changes due to spontaneous recovery (Dalton et al., 2024), and long-term changes over time (Kim et al., 2022). This feature of the CoreLex analysis is especially useful as confrontation naming tests may not be sensitive in indexing minute changes in word-finding and other language abilities in PWA. Finally, there is empirical evidence to support concurrent validity of core lexicon scores with standardized assessments, as well as other discourse measures (Dalton et al., 2020a; Dalton et al., 2024; Kim and Wright, 2020).

Using a similar approach of CoreLex analysis, Fromm et al. (2024) used language samples from Cookie Theft picture descriptions to evaluate potential differences in typical language use across three groups of participants: adults diagnosed with AD (ProbableAD), and adults with cognitive decline (MCI), and age-matched adults without cognitive impairments (controls). They identified significant differences in the use of core lexicon words across the groups. When compared to adults with MCI and control participants, adults with ProbableAD produced significantly fewer core lexicon words overall and produced them significantly later than the other two groups. While the ProbableAD and MCI groups did not differ significantly in the use of core lexicon function words, both groups produced significantly fewer core lexicon content words (nouns and verbs) compared to control participants. Comparative analyses of core lexicon word production across the Cookie Theft picture quadrants revealed that all the groups produced a core lexicon word within the top-left quadrant during the initial stages of the picture description task. However, the ProbableAD group was significantly slower in producing core lexicon words compared to the other two groups for the remaining three quadrants. The authors attributed these group differences in visuospatial patterns to a combination of four factors. First, the main action in the picture (boy taking the cookies out of the cookie jar) was depicted in the top-left quadrant. Second, it was the only quadrant with a written world label (COOKIE JAR). Third, the line drawing within the top-left quadrant was relatively simple. Fourth, English speakers tend to intuitively look toward the top-left quadrant during scene perception.

Understanding these visuospatial patterns while engaged in a discourse production task, such as describing the Cookie Theft picture, can provide valuable insights into our understanding of visuospatial and language processing in PWA and their potential interrelationships. Further, the quadrant-based method from Fromm et al. (2024) used in older adults with cognitive decline is particularly relevant to the current study, as it provides a direct precedent for analyzing visuospatial patterns during discourse production tasks in PWA. It is possible that Fromm et al. (2024) findings may be confounded by stimulus-specific properties, raising questions about whether quadrant-based differences reflect true cognitive-linguistic processes or features of the picture itself. A direct measurement of visuospatial behavior using eye-tracking methods may hold the answers to this question. The current study builds upon Fromm et al.’s approach by using a different picture stimulus *(Cat Rescue)* which may limit quadrant-specific biases by distributing salient elements more evenly across the image and by systematically examining how visuospatial attention and language production interact in PWA.

## 2 Current study

We extend the above analysis and apply it to individuals with stable, post-stroke aphasia, comparing the timing, order, and spatial location of CoreLex production as a proxy for visuospatial processing during a picture scene narrative task *(Cat Rescue)* with that of HCP without brain injuries. We hypothesize that investigating the order and timing of CoreLex productions with respect to the spatial location of the CoreLex items in the picture will provide insights into how HCP and PWA use the visual stimulus to support completion of a picture description task. For example, order and timing of production may yield information about whether individuals scan an image from left to right, top to bottom, or some other pattern. Identifying similarities and/or differences between HCP and PWA may allow us to identify underlying strengths that can be leveraged to enhance speech language therapy, or weaknesses that should be addressed to maximize the effect of therapy. We also investigate the impact of aphasia severity on the timing and order of production of CoreLex items. There is ample research demonstrating differences in language processing and production across aphasia types and severities, as such, identifying whether overall patterns of similarities and differences are consistent across the severity spectrum will allow for more targeted application of these results and planning for future research.

Since previous research examining CoreLex production between PWA and HCP has demonstrated significant group differences (e.g., Dalton et al., 2020b; Kim et al., 2019), an investigation of this nature may provide additional insight into group differences in CoreLex production. Picture scene narrative tasks, such as the *Cat Rescue* task from the AphasiaBank protocol, depict real-world scenes while providing less structure regarding the correct sequencing or organization of a story than picture sequence tasks (e.g., where a panel of four to six images provide the sequence of actions in a story Capilouto et al., 2005). As such, visuospatial processing is likely to play a greater role in discourse production of a picture scene than a picture sequence. Importantly for this study, CoreLex items are relatively evenly distributed across the *Cat Rescue* picture, allowing for this analysis. In the current study, we leverage the AphasiaBank database, a repository of discourse samples from individuals with and without brain injury. Our goal is to investigate group differences in the timing, order, and spatial location of core lexicon items produced while completing the *Cat Rescue* task. Demonstrating the feasibility of these measures and establishing a baseline will allow for future investigations examining change over time or in response to treatment (especially those treatments which contain a visuospatial working memory component) that may yield insights beyond isolated language and visuospatial tasks. For this project, we aimed to:

1. Determine group differences in the production of core lexicon items between PWA and HCP as well as the impact of aphasia severity on core lexicon production.
2. Determine group differences in the elapsed time to produce the first core lexicon content word in each quadrant between PWA and HCP;
3. Determine group differences in the timing and order of production of each core lexicon content word in each quadrant between PWA and HCP; and.
4. Describe the impact of aphasia severity on the elapsed time to produce the first core lexicon content word in each quadrant as well as the timing and order of production of each core lexicon content word.

## 3 Methods

### 3.1 AphasiaBank database

The AphasiaBank database is the largest repository of discourse produced by individuals with aphasia (MacWhinney et al., 2011), containing more than 600 samples from PWA across an array of discourse tasks. The database also includes over 350 discourse samples from neurotypical control participants, making it possible to compare groups and obtain additional insights on the impact of stroke on functional communication. The set of discourse samples within the database has been contributed by numerous researchers across the United States and internationally. The AphasiaBank database uses a standardized protocol to elicit discourse samples, including a picture scene narrative, familiar narrative retell, procedural task, picture sequence narratives, and personal event narratives. All transcripts in the database have been transcribed in CHAT format (Codes for the Human Analysis of Transcripts) and validated, automated analysis of core lexicon checklists can be completed using the freely available CLAN (Computerized Language ANalysis) software (Dalton et al., 2024). These checklists were developed by identifying the lexical items used by at least 50% of a normative sample of healthy control participants and include both content and function words. In addition, most transcripts have been linked to the associated media file (audio or video) which allows for timing of speech and non-speech behaviors with millisecond precision.

### 3.2 Participant description

#### 3.2.1 Control participants

A total of 386 English-language transcripts from HCP were accessed from the following corpora: Baycrest (Kielar et al., 2016), Capilouto, Kempler, MSU, NEURAL, NEURAL-2, Richardson, UMD (Faroqi-Shah and Milman, 2018), UNH, and Wright corpora. Of these, transcripts were excluded if the individual did not complete the *Cat Rescue* task or the transcript was not linked to the associated media file for timing word productions (*n = 31*), and if the transcript was not from the first data collection timepoint (*n = 56*). We excluded transcripts from later temporal intervals to limit any overrepresentation of a single individual’s data in the sample. Following this process, a total of 299 transcripts from HCP were included, with at least one speaker representing each of the above corpora. Transcripts were not excluded if demographic or testing data was missing. HCP included 174 females and 125 males, with an average age of 57 (*SD* = 21) and an average education of 16 years (*SD* = 2.7). A summary of the demographic information for HCP is provided in Table 1, including age, education, sex, and race/ethnicity. In terms of language backgrounds, the transcripts included discourse samples from monolingual speakers (*n* = 208), multilingual speakers (*n* = 11), late bilingual speakers (*n* = 12), and a childhood bilingual speaker (*n* = 1). A total of 67 HCP participants’ demographic data records did not include information on language background.

**Table 1:** A summary of the participant demographic information for HCP and PWA, as well as the aphasia severity groups.

Participants	Age (yrs)			Education (yrs)			Sex		Race/ethnicity								
	Mean (SD)	Range	MIS	Mean (SD)	Range	MIS	F	M	AA	AI	AS	HL	MIX	NH	OTH	WH	MIS
HCP (*N* = 308)	56 (21)	19–90	1	18 (2.6)	10–25	12	185	133	9	-	3	9		-	-	260	37
PWA* (*N* = 384)	62 (12)	25–91	2	16 (3)	7–25	17	154	230	39	1	5	6	4	2	2	310	15
Latent (*N* = 55)	59 (14)	25–80	-	17 (3.5)	10–24	2	32	23	3	-	1	2	1	1	1	40	6
Mild (*N* = 134)	62 (12)	31–86	1	16 (3)	12–25	5	50	84	9	-	2	2	-	-	1	115	5
Moderate (*N* = 130)	61 (12)	26–91	-	15 (3)	7–25	5	55	75	19	1	2	2	2	1	-	101	2
Severe (*N* = 44)	63 (13)	25–83	-	15 (3)	8–20	4	13	31	7	-	-	-	1	-	-	35	1
Profound (*N* = 10)	69 (9)	56–85	-	17 (3)	12–23	-	0	10	-	-	-	-	-	-	-	10	-

AA, African American; AI, American Indian; AS, Asian; F, Female; HL, Hispanic or Latino; M, Male; MIS, Missing; MIX, Mixed; NH, Native Hawaiian; OTH, Other; WH, White. * This group includes 11 individuals who are missing aphasia severity data and are excluded from the severity sub-group analysis.

#### 3.2.2 Aphasia participants

A total of 641 English-language transcripts of PWA were accessed from the following corpora: ACTW, Adler, APROCSA (Ezzes et al., 2022), Baycrest (Kielar et al., 2016), BU, CMU, Elman (2016), Fridriksson, Garrett, Kansas, Kempler, Kurland, MSU, NEURAL, NEURAL-2, Richardson, SCALE, STAR, TAP, TCU, TCU-bi, Thompson, Tucson, UCL (Dean, 2021), UMD (Faroqi-Shah and Milman, 2018), UNH, Whiteside, Williamson, Wozniak, and Wright. Of these, transcripts were excluded if: (a) the individual had an unknown or non-stroke aphasia etiology (*n = 16*), (b) the individual did not complete the *Cat Rescue* task or the transcript was not linked to the associated media file for timing word productions (*n = 47*), or (c) if the transcript was not from the first data collection timepoint (*n = 194*). This step resulted in the inclusion of a total of 384 transcripts from speakers with aphasia, with at least one speaker retained from each of the above corpora. Similar to HCP transcripts, aphasia transcripts with missing demographic or testing data were not excluded. PWA included 154 females and 230 males, with an average age of 61 (*SD* = 12) and an average education of 16 years (*SD* = 3). A summary of the demographic information for participants across aphasia subtypes is provided in Table 1, and aphasia severity, time post stroke, and motor speech diagnoses are provided in Table 2. The sample of PWA (*N* = 384) included discourse samples from monolingual speakers (*n* = 334), multilingual speakers (*n* = 9), late bilingual speakers (n = 13), and childhood bilingual speakers (*n* = 18), A total of 26 transcripts were missing this information.

**Table 2:** Language specific demographic data for participants across aphasia severity groups.

Aphasia severity (MIS = 11)	WAB-R AQ		Aphasia duration (yrs)		Motor speech diagnosis	
	Mean (SD)	Range	Mean (SD)	Range	AOS	Dysarthria
Latent Aphasia (*N* = 55)	96.5 (1.8)	93.8–100	5 (3.4)	1–15.7	6	2
Mild Aphasia (*N* = 134)	85.5 (5.7)	75–93.5	5.6 (6)	0.08–44	28	11
Moderate Aphasia (*N* = 130)	64 (7.1)	50–74.9	6.3 (5.9)	0.45–32.2	49	13
Severe Aphasia (*N* = 44)	40.5 (6.8)	27.3–49.5	3.6 (4.4)	0.2–25	17	1
Profound Aphasia (*N* = 10)	19.2 (3.8)	10.8–24.5	5.9 (6.6)	0.7–20	9	3

AOS, Apraxia of Speech; MIS, Missing; WAB-R AQ (Western Aphasia Battery - Revised Aphasia Quotient).

In terms of overall language assessment results, WAB-R AQ (Western Aphasia Battery – Revised Aphasia Quotient) scores were computed from the sample (*M* = 72.8; *SD* = 20.2; Range = 10.8–100). This information was missing in 11 transcripts. Additionally, the duration of aphasia (in years) was extracted for PWA (*M* = 6; *SD* = 5; Range = 0–44). A total of 49 transcripts were missing this information. An overview of the language assessment results including WAB-R AQ, aphasia duration, and concomitant motor speech diagnosis across aphasia subtypes is also provided in Table 2. Aphasia severity was based on the WAB-R AQ, which includes measures of language production and comprehension. Severity was interpreted in accordance with the severity ratings in the scoring manual (Kertesz, 2007): mild (<93.8), moderate (50–74), severe (25–49), and profound (0–24). Additionally, we included the sub-group of individuals who score above the WAB-R AQ cut-off (>93.8), but who have experienced a stroke and continue to report communication impairments that impact everyday activities. These individuals have been described elsewhere in the literature as producing discourse that is significantly different from HCP and from individuals with clinical aphasia (Cunningham and Haley, 2020; Dalton and Richardson, 2015; Dalton and Richardson, 2019; DeDe and Salis, 2020; Fromm et al., 2017; Martzoukou et al., 2023; Richardson et al., 2018; Richardson et al., 2021; Salis and DeDe, 2022). While this group of individuals has previously been referred to as “not aphasic by WAB” in much of the literature, we refer to them here as having “latent” aphasia, to reflect the fact that identification is not tied to a particular standardized assessment, but rather to the residual, underlying language impairment that may not be immediately visible, but which becomes apparent under specific conditions. Individuals with missing WAB-R AQ scores were excluded from the severity sub-group analyses but included in the overall PWA group analyses.

### 3.3 Procedures

**Figure 1:**
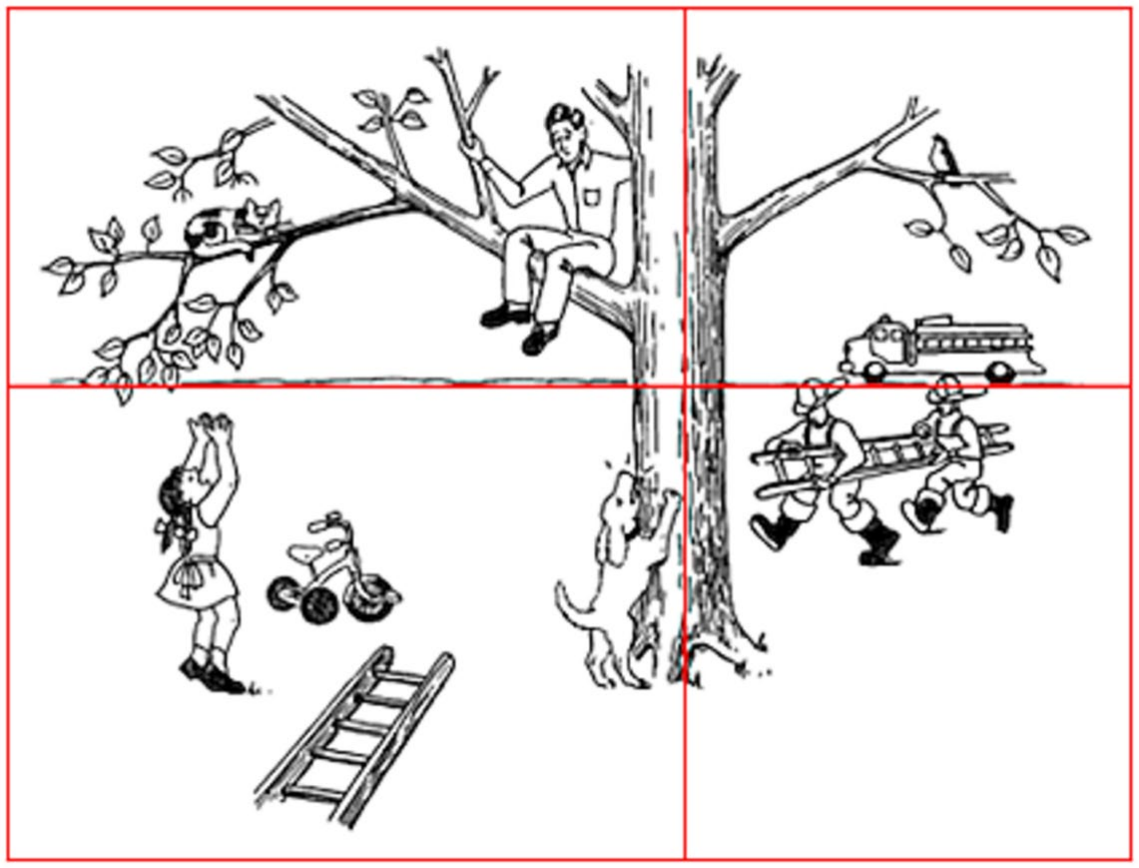
Quadrants developed for the *Cat Rescue* picture (Nicholas and Brookshire, 1993).

For the current study, we used the picture scene of *Cat Rescue* from Nicholas and Brookshire (1993). The original picture, along with overlaid boundaries distinguishing the four quadrants is provided in Figure 1. Participant instructions for eliciting this task were: “Take a look at the picture. It tells a story. Please tell me the story with a beginning, middle, and end. You can look at the picture as you tell the story.” Previous research has demonstrated that these instructions are more likely to elicit discourse with greater complexity and increased informativeness compared to instructions that ask the participant to describe or “talk about” an image (Wright and Capilouto, 2009).

Transcripts from the AphasiaBank database were extracted in CHAT (Codes for the Human Analysis of Transcripts) format. Core lexicon scores and timing data were automatically retrieved using the CLAN program. To ensure that accurate core lexicon scores were calculated for study Aim 1, we followed the procedure outlined by Dalton et al. (2024) and used the following three CLAN commands:

1. chstring +q1 *.cha: modified transcripts to ensure that speaker’s actual productions were scored rather than transcriber-identified targets during paraphasias; to remove revision codes; and to remove underscores from “frozen” phrases (e.g., “you know”) so that all words were counted.
2. mor *.chstr.cex: re-ran the morphological command to tag and disambiguate the morphological function of newly analyzable words due to the chstring command.
3. corelex +lcat +t*par *.chstr.cex: used the morphological tier to search for the list of core lexicon lemmas in the *Cat Rescue* checklist and returned an excel spreadsheet with all participants’ core lexicon productions and core lexicon score.

In order to analyze the timing of core lexicon production for study Aims 2–4, we calculated the time elapsed from the start of the task to the first production of each core lexicon word (in ms) by using a new CLAN command (corelex +lcat-short +t*par +w *.cha). This command was programmed to search the word alignment tier (%wor) (Liu et al., 2023) of included transcripts and provide the output of the time stamp of *Cat Rescue* CoreLex checklist items present in the transcript. This step was completed for each participant. If a CoreLex item was not present in the transcript, then the CLAN output listed “NA” in that cell. Given that only the first production of each CoreLex item was scored and timed, any perseveration errors present in the transcripts were not expected to impact the data. Importantly, the timing command only searched for core lexicon content words (bark, call, cat, climb, department, dog, father, fire, girl, ladder, stuck, tree, and fireman), as the timing of function words would likely not be localized to a specific quadrant of the picture, nor would we expect the production of functors to be particularly informative given their high frequency. To determine order of CoreLex production, we calculated the median time elapsed from the start of the task to production of each CoreLex item for each group (or, said another way, the point at which 50% of the group produced the CoreLex item). The median times were then ranked from shortest to longest for HCP and PWA, and by aphasia severity groups.

For Aim 2, each core lexicon content item was assigned a quadrant for analysis in order to improve the granularity of results (see Figure 1): top left (cat, climb, father, stuck); top right (fire, department); bottom left (girl, ladder); bottom right (bark, dog, fireman). Exceptions were the items “tree,” since it extended across all four quadrants; and “call,” since this action is not depicted in the image. These items were excluded from the analyses. While “climb” can be used to describe the actions of both the cat and the father, they are in the same quadrant (top left); therefore, “climb” was included as a content item in that quadrant. Although a ladder is pictured in the bottom left and bottom right quadrants of the image, we assigned “ladder” to the bottom left quadrant based on the expected story sequence (e.g., the ladder must fall in the bottom left quadrant in order to precipitate the firemen arriving with their ladder).

### 3.4 Data analysis

All analyses were completed using SPSS v29. Prior to conducting the planned analyses, groups were compared on the demographic variables of age, years of education, and sex. Independent samples t-tests revealed significant group differences in age (*t* = −3.559, *p* < 0.001, *g* = −0.292) and sex (χ^2^ = 22.04, *p* < 0.001, ϕ = −0.18). The HCP group was younger and included more males as compared to the PWA group. No significant difference was observed between groups on years of education (*t* = 0.398, *p =* 0.345, *g* = −0.031). While age and sex differ significantly between groups, the effect size for both variables was small, indicating the difference likely does not hold any practical significance.

To address Aim 1, a one-way ANOVA (group x CoreLex score) was calculated with planned *post hoc* testing using Fischer’s least significant difference (LSD) to identify pairwise differences between HCP and PWA across the severity spectrum. For Aims 2–4, survival analyses were completed to examine how many individuals in each group produced individual core lexicon words (e.g., the frequency of production for the group) and the amount of time that elapsed between story start and core lexicon word production (also known as the hazard rate). Traditionally, survival analyses are used to compare the average time to an event occurring among two or more groups. For example, a survival analysis might be used to compare the time to cancer recurrence in two groups of cancer patients receiving different treatments. For patients who experienced a recurrence, the time to event would be the number of days, months, or years that elapsed before the cancer recurred. For patients who do not experience a recurrence, the time would be the duration of the study period. An important component of this analysis is that all individuals who did not experience the “event” (in our case, production of a CoreLex item) should have the same endpoint. Therefore, the longest story duration present in the dataset was entered as the endpoint for individuals who did not produce a given CoreLex item (e.g., all cells with “NA” as the CLAN output).

Kaplan–Meier survival curves obtained from this analysis were used to depict the time distribution (*t*) until a core lexicon word was spoken. Additionally, Kaplan–Meier estimates were utilized to illustrate the time distribution (*t*) of core lexicon word production across participant groups. For ease of interpretation, we plotted the complement of the standard Kaplan–Meier curve by noting the proportion of participants within each group who had produced a specific word of interest (x-axis) by time *“t”* (y-axis). While these complementary plots are known as “1-survival” curves, we refer to them as Kaplan–Meier curves for simplicity and clarity. These plots are used to compare group differences in the timing of production of a specific word of interest. We applied the log-rank test to further evaluate if the group differences in the timing of core lexicon production were statistically significant. To answer Aim 2, we first grouped the CoreLex items in each quadrant together and then identified the shortest time to produce any CoreLex item in that quadrant. Using this method, it is possible that the times for different individuals were associated with production of different CoreLex items. For example, some individuals might have produced “cat” first, while others may have produced “father” first. Regardless of CoreLex item, the shortest elapsed time was used in this analysis. To answer Aim 3, we examined the timing of each CoreLex content word separately, regardless of quadrant in which it was located. Finally, to answer Aim 4, we used the same procedures as in Aim 2 and 3 and completed pairwise log-rank comparisons between HCP and sub-groups of PWA with varying severity.

## 4 Results

### 4.1 Group differences in CoreLex production

The results of the one-way ANOVA revealed a significant effect of group on CoreLex production (F[5,666] = 181.842, *p* < 0.001, η^2^ = 0.577), wherein HCP produced significantly more CoreLex items than PWA. *Post hoc* LSD pairwise comparisons to investigate the effect of aphasia severity on these results demonstrated that HCP also produced significantly more CoreLex items than all aphasia severity groups, including individuals with latent aphasia (see Table 3 for full ANOVA results).

**Table 3:** Results of the one-way ANOVA and post-hoc pairwise comparisons investigating difference in CoreLex scores between HCP and PWA, and among HCP and individuals with varying severity levels.

Core lexicon score	HCP	PWA	Latent	Mild	Moderate	Severe	Profound		Post-hoc		
									Group (HCP v Severity)	Mean Difference	*p*
	M (SD)	M (SD)	M (SD)	M (SD)	M (SD)	M (SD)	M (SD)	F[5,666] (η^2^)			
	25.5 (4)	16.9 (7.6)	23.6 (4)	20 (5)	14.5 (6)	8.3 (6.6)	3 (4.9)	181.842* (0.577)	Latent	1.9	0.008
									Mild	5.6	< 0.001
									Moderate	11	< 0.001
									Severe	17.2	< 0.001
									Profound	22.5	< 0.001

* *p* < 0.001.

### 4.2 Production of first CoreLex item by quadrant

**Figure 2:**
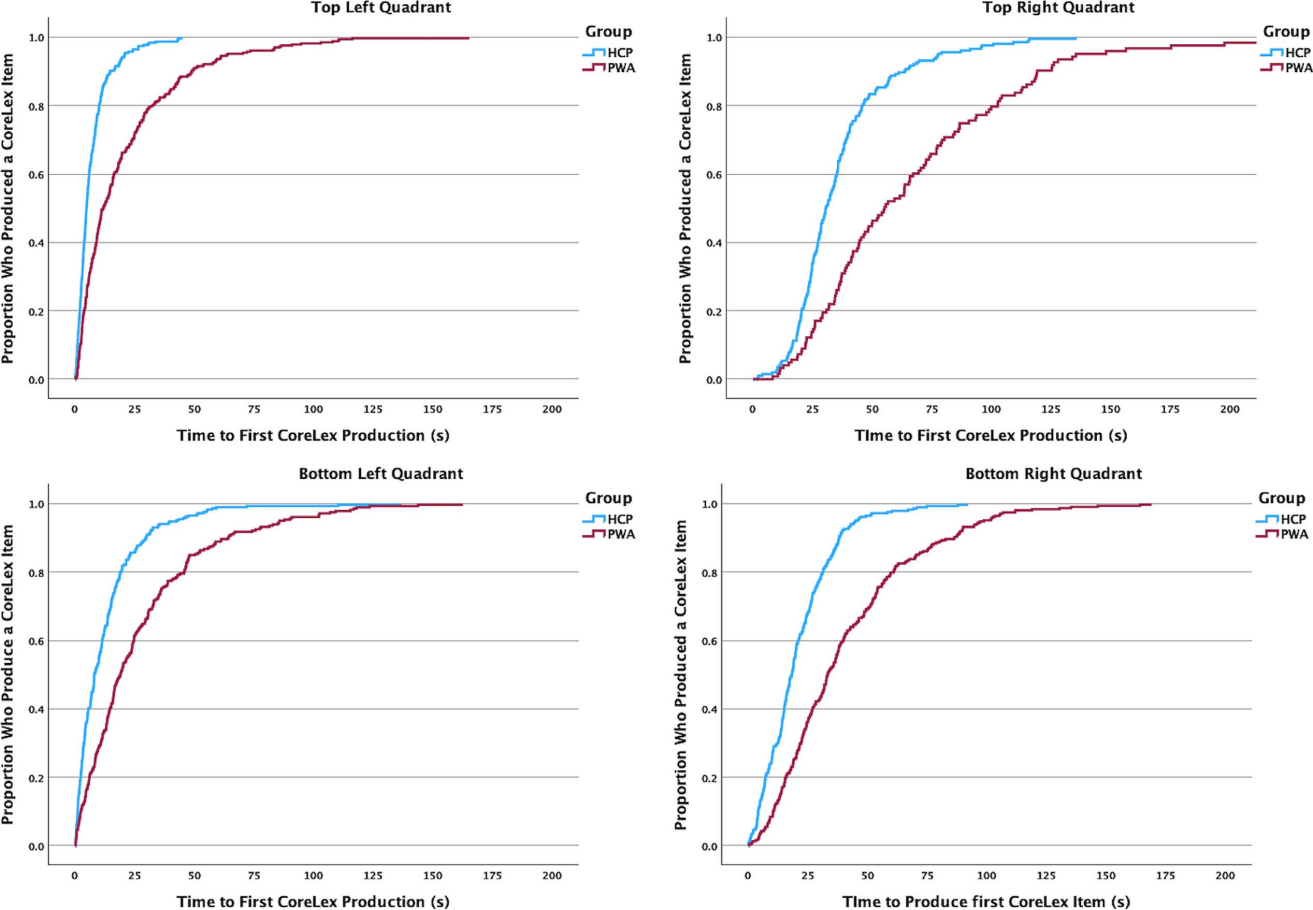
Kaplan-Meyer survival curves of time required to produce a CoreLex item in each quadrant for HCP and PWA.

Across all four quadrants, HCP were significantly faster in producing any CoreLex item compared to PWA (TL: χ^2^ = 129.164, *p* < 0.001; BL: χ^2^ = 119.693, *p* < 0.001; BR: χ^2^ = 70.537, *p* < 0.001; TR: χ^2^ = 64.09, *p* < 0.001). Based on the median time to production (e.g., the time at which half the group had produced the first CoreLex item for a given quadrant), HCP first spoke about the *top left* quadrant (median = 4.7 s), followed quickly by the *bottom left* quadrant (median = 8.1 s). There was a longer delay in producing a CoreLex item in the *bottom right* quadrant (median = 18.3 s). The first CoreLex item in the *top right* quadrant was typically produced last (median = 30.5 s). PWA followed the same pattern of production across quadrants, however after a much longer elapsed time compared to HCP (see Table 4 and Figure 2). The median time to produce a CoreLex item in the *top left* quadrant for PWA was 11.8 s, followed by 19 s to produce an item in the *bottom left* quadrant, 33 s to produce an item in the *bottom right* quadrant, followed by 55.2 s to produce an item in the *top right* quadrant.

**Table 4:** Results of the χ^2^ tests of the difference in survival curves between HCP and PWA for each core lexicon item (listed in order of HCP production).

Core lexicon word	Percentage of group producing item		Median production time (s)		Median time difference	Log-rank χ^2^
	HCP	PWA	HCP	PWA		
Cat	97.7	82	5.185	13.368	−8.183	164.375**
Girl	74.7	55.2	6.482	16.543	−19.143	57.395**
Father	71.4	34.9	13.3385	24.117	−10.7785	131.665**
Climb	61.7	19.5	15.379	22.96	−7.581	150.808**
Stuck	50.3	16.9	17.053	27.345	−9.89	97.598**
Dog	87.7	72.1	17.7035	36.758	−19.0545	97.505**
Ladder	83.8	53.4	21.734	40.877	−10.292	141.375**
Bark	46.4	39.3	27.1065	43.18	−16.0735	8.603*
Fire	65.9	31.5	30.645	55.189	−24.544	114.299**
Department	55.8	13.3	30.7025	44.715	−14.0125	158.845**
Fireman	38	44.3	32.5905	54.39	−10.061	0.373
Top left			4.7	11.8	−7.1	129.164**
Bottom left			8.1 (−2.4)	19 (−7.2)	−10.9	119.693**
Bottom right			18.3 (−10.2)	33 (−14)	−14.7	70.537**
Top right			30.5 (12.2)	55.2 (−22.2)	−24.7	64.09**

The percentage of each group who produced the CoreLex item, and the median elapsed time to produce each item by group is reported, along with the difference in median elapsed time between groups (calculated as HCP minus PWA). *p < 0.01, **p < 0.001.

### 4.3 Timing and order of production for each CoreLex item

Kaplan–Meier survival analyses demonstrated significant differences in the timing of CoreLex production between HCP and all PWA across most CoreLex items. Further, HCP demonstrated a shorter time to CoreLex word production compared to PWA.

#### 4.3.1 Timing of production

**Figure 3:**
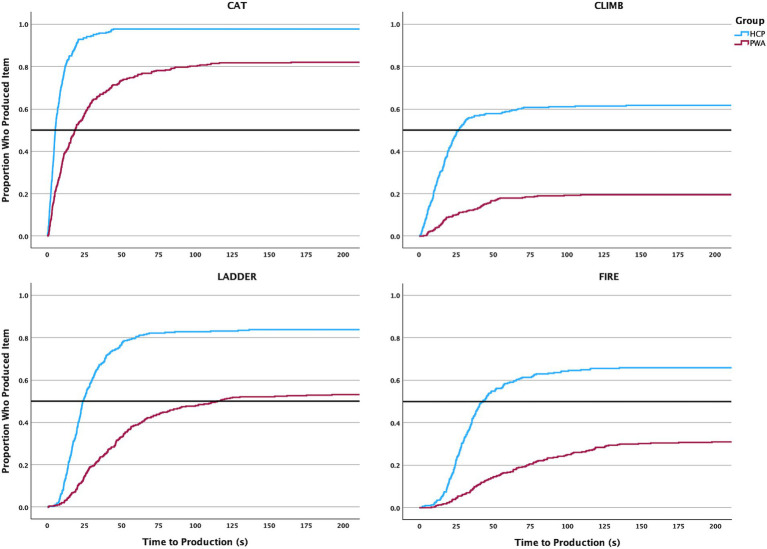
Select, illustrative Kaplan-Meyer survival curves of time required to produce CoreLex items for HCP and PWA.

HCP demonstrated an overall shorter time to production for the CoreLex items “bark,” “cat,” “climb,” “department,” “dog,” “father,” “fire,” “girl,” “ladder,” and “stuck” (see Table 4). Review of the Kaplan–Meier survival curves for CoreLex items in the *top left* quadrant (See Figure 3) shows that HCP produced the word “cat” early in the story, with 50% of controls saying the word within approximately 5 s of beginning the task, and nearly all HCP producing the word. PWA produced “cat” much later, with 50% saying the word within approximately 18 s of beginning the task. However, a similar proportion of PWA as HCP did eventually produce the word. In contrast, while ~70% of HCP eventually produced the word “father,” only about 45% of PWA produced it. Finally, between 50 and 60% of HCP said “climb” and “stuck,” but only about 20% of PWA produced these items, and they were produced less quickly by PWA than HCP. Survival curves for CoreLex items in the *bottom left* quadrant show that half of HCP produced “girl” by approximately 10 s in the task, while half of PWA had not produced “girl” until 50 s had elapsed. “Ladder” was produced later in the story, with half of HCP producing the word by 20 s, and half of PWA producing the word by 75 s. A similar proportion of participants in each group produced the two items (~55% of PWA; ~80% of HCP). When examining the survival curves for items in the bottom right quadrant, both groups produced “dog” in the shortest median time, with half of HCP producing “dog” within 20 s. For both groups, the median time to produce “bark” was much longer than “dog” (HCP = 45 s; PWA = 120 s). “Fireman” showed a unique pattern of production compared to other CoreLex items. While the median time to production for HCP was shorter than PWA (HCP = 60s; PWA = 105 s), a slightly higher proportion of PWA eventually produced “firemen” than HCP. This was also the only survival curve for which significant differences between groups were not observed. Finally, when examining the survival curves for items in the *top right* quadrant, “fire” had a shorter mean time to production (43 s) than “department” (60s) for HCP. While 66 and 56% of all HCP eventually said the words “fire” and “department,” respectively, only 31 and 14% of PWA produced them.

Given the significant differences between groups on demographic variables, and the potential influence of age and education on word-finding, we also conducted a Cox Regression analysis, which extends the Kaplan–Meier analysis by separating out the unique contribution of multiple variables to group differences. We entered group, age, and education into the regression analysis. Results showed that group status (e.g., HCP vs. PWA) was a significant predictor of production time for all CoreLex items except “firemen,” even after controlling for age and education (Table 5), replicating the Kaplan–Meier findings.

**Table 5:** Cox regression modeling hazard ratios with 95% confidence intervals for differences between HCP and PWA in the time to production of core lexicon items, controlling for age and education.

Core lexicon item	Hazard ratio (95% CI)
Bark	0.706 (0.557–0.894) *p* = 0.004
Cat	0.348 (0.292–0.414) *p* < 0.001
Climb	0.217 (0.164–0.288) *p* < 0.001
Department	0.174 (0.126–0.239) *p* < 0.001
Dog	0.418 (0.349–0.499) *p* < 0.001
Father	0.274 (0.219–0.344) *p* < 0.001
Fire	0.305 (0.241–0.386) *p* < 0.001
Fireman	1.015 (0.796–1.295) *p =* 0.903
Girl	0.498 (0.410–0.605) *p* < 0.001
Ladder	0.322 (0.264–0.391) *p* < 0.001
Stuck	0.292 (0.215–0.397) *p* < 0.001
Left side	0.366 (0.308–0.435) *p* < 0.001
Top-left quadrant	0.318 (0.266–0.380) *p* < 0.001
Bottom-left quadrant	0.414 (0.347–0.494) *p* < 0.001
Right side	0.309 (0.258–0.369) *p* < 0.001
Top-right quadrant	0.308 (0.244–0.389) *p* < 0.001
Bottom-right quadrant	0.433 (0.364–0.515) *p* < 0.001

Hazard ratios show significant between group differences for all comparisons except the item “fireman”.

#### 4.3.2 Order of production

**Figure 4:**
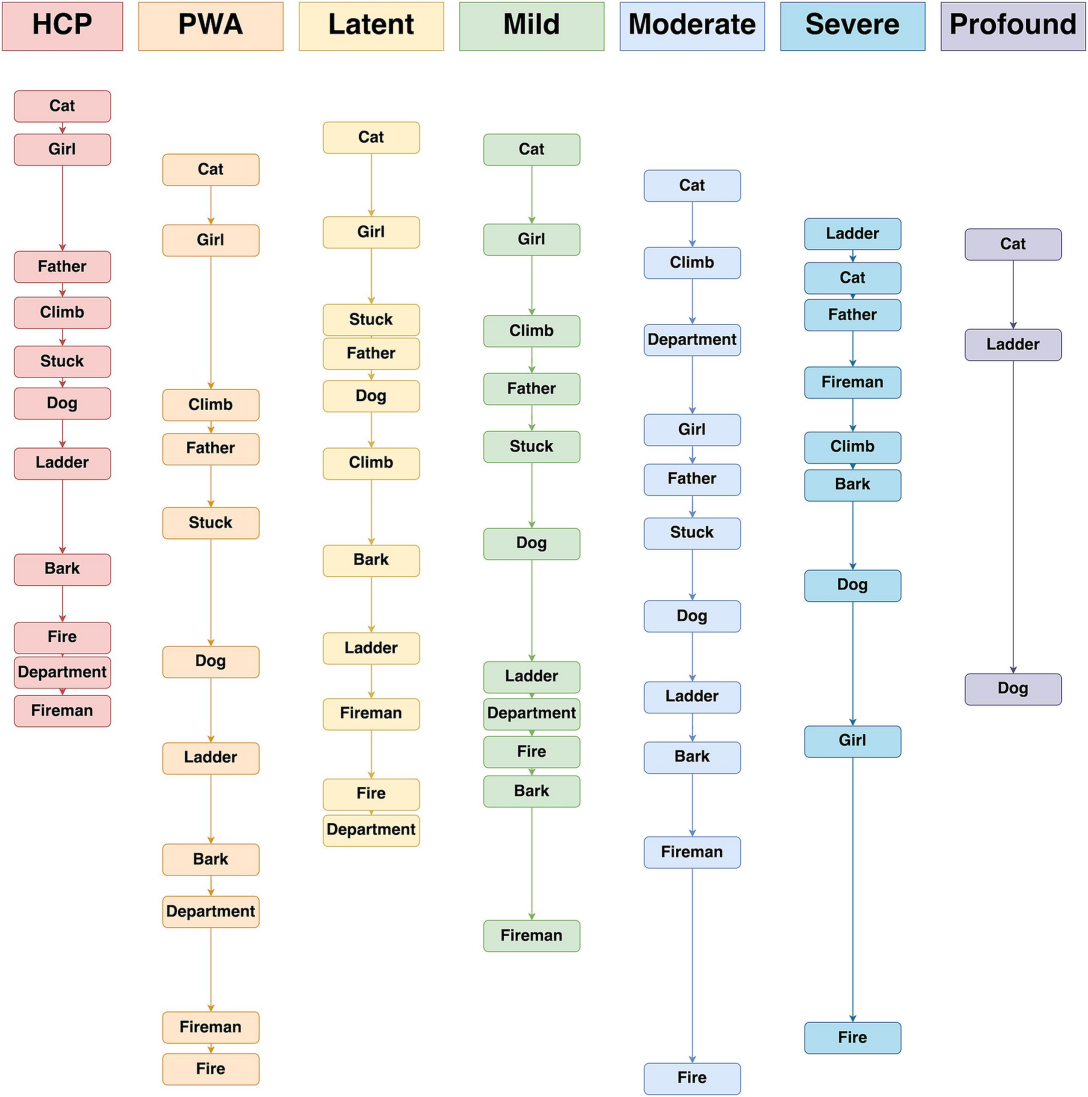
Order of production of CoreLex words by group, based on the median time to produce each item. A larger vertical distance between the group label and the first CoreLex item reflects a longer time taken to produce that first item. Similarly, vertical distance between words indicates the relative timing of words, with larger distances indicating increased time between production of one CoreLex item and the next.

In contrast to Aim 1, wherein PWA and HCP introduced the quadrants in the same order, there were some discrepancies to the order in which CoreLex items were introduced (based on group median times; see the first two columns in Figure 4). First, HCP produced “father” before “climb,” while PWA produced “father” after “climb.” Second, HCP produced “fire,” “department,” and then “fireman” (in that order) as the last three CoreLex items, while the final three CoreLex items for PWA were “department,” “firemen,” and then “fire.” All other CoreLex items were produced in the same order by both PWA and HCP.

### 4.4 Impact of aphasia severity

#### 4.4.1 Time of Core lexicon production

##### 4.4.1.1 Latent aphasia

**Figure 5:**
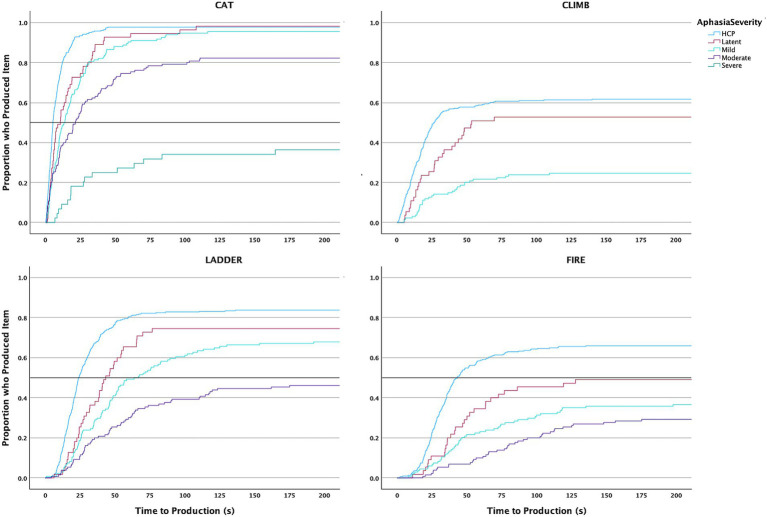
Select, illustrative Kaplan-Meyer survival curves of time required to produce CoreLex items for HCP and PWA severity groups.

In the *top left* quadrant, the first CoreLex item was produced by half of the individuals with latent aphasia by 7.2 s (χ^2^ = 18.261, *p* < 0.001). In the *bottom left* quadrant, the first CoreLex item was produced by half of individuals with latent aphasia by 14.2 s (χ^2^ = 6.755, *p* = 0.009). In the *bottom right* quadrant, the first CoreLex item was produced by half of individuals with latent aphasia by 21.7 s (χ^2^ = 11.65, *p* < 0.001). In the *top right* quadrant, the first CoreLex item was produced by half of individuals with latent aphasia by 41.8 s (χ^2^ = 7.399, *p* = 0.007). When comparing the survival curves of HCP and individuals with latent aphasia for production of each CoreLex item, significant differences were seen for production of “cat,” “department,” “father,” “fire,” ““fireman,” “ladder,” and “stuck” (see Table 6, Figure 5). For all items, individuals with latent aphasia demonstrated a longer median production time than HCP.

**Table 6:** Results of log-rank χ^2^ tests examining differences in survival curves between HCP and the aphasia severity groups.

Core lexicon item	Latent aphasia			Mild aphasia			Moderate aphasia			Severe aphasia		
	Log-Rank χ^2^	*p*	Time difference (s)	Log-Rank χ^2^	*p*	Time difference (s)	Log-Rank χ^2^	*p*	Time difference (s)	Log-Rank χ^2^	*p*	Time difference (s)
Bark	3.23	0.072	+7.30	0.034	0.855	+18.89	12.59	<0.001*	+21.89	-	-	-
Cat	10.58	0.001*	+4.43	42.57	<0.001*	+6.58	82.460	<0.001*	+9.45	100.86	<0.001*	+17.48
Climb	3.27	0.07	+11.21	51.95	<0.001*	+7.43	-	-	-	-	-	-
Department	8.85	0.003*	+11.54	48.19	<0.001*	+14.04	-	-	-	-	-	-
Dog	2.30	0.13	+4.59	26.36	<0.001*	+18.22	59.62	<0.001*	+22.51	46.28	<0.001*	+19.36
Father	6.67	0.01*	+7.30	38.06	<0.001*	+11.45	86.00	<0.001*	+17.24	39.62	<0.001*	+9.90
Fire	7.79	0.005*	+11.19	25.57	<0.001*	+14.74	57.82	<0.001*	+47.22	-	-	-
Fireman	4.01	0.045*	+6.81	7.18	0.007*	+8.00	0.06	0.805	+21.56	-	-	-
Girl	0.45	0.504	+13.97	5.44	0.02*	+22.40	45.89	<0.001*	+24.21	-	-	-
Ladder	8.86	0.003*	+3.41	36.64	<0.001*	+11.15	81.88	<0.001*	+16.42	-	-	-
Stuck	5.83	0.016*	+2.86	29.71	<0.001*	+7.29	52.90	<0.001*	+20.64	-	-	-

*Indicates statistically significant difference.

##### 4.4.1.2 Mild aphasia

In the *top left* quadrant, the first CoreLex item was produced by half of individuals with mild aphasia by 10.5 s (χ^2^ = 59.343, *p* < 0.001). In the *bottom left* quadrant, the first CoreLex item was produced by half of individuals with mild aphasia by 15.2 s (χ^2^ = 23.382, *p* < 0.001). In the *bottom right* quadrant, the first CoreLex item was produced by half of individuals with mild aphasia by 32.8 s (χ^2^ = 68.169, *p* < 0.001). In the *top right* quadrant, the first CoreLex item was produced by half of individuals with mild aphasia by 44.7 s (χ^2^ = 26.372, *p* < 0.001). When comparing the survival curves of HCP and individuals with mild aphasia for production of each CoreLex item, significant differences were seen for production of “cat,” “climb,” “department,” “dog,” “father,” “fire,” ““fireman,” “girl,” “ladder,” and “stuck” (see Table 6). For all items, individuals with mild aphasia demonstrated a longer median production time than HCP.

##### 4.4.1.3 Moderate aphasia

In the *top left* quadrant, the first CoreLex item was produced by half of individuals with moderate aphasia by 14.5 s (χ^2^ = 74.043, *p* < 0.001). In the *bottom left* quadrant, the first CoreLex item was produced by half of individuals with moderate aphasia by 28.4 s (χ^2^ = 62.382, *p* < 0.001). In the *bottom right* quadrant, the first CoreLex item was produced by half of individuals with moderate aphasia by 37.9 s (χ^2^ = 81.579, *p* < 0.001). In the *top right* quadrant, the first CoreLex item was produced by half of individuals with moderate aphasia by 77 s (χ^2^ = 42.878, *p* < 0.001). When comparing the survival curves of HCP and individuals with mild aphasia for production of each CoreLex item, significant differences were seen for production of “bark,” “cat,” “dog,” “father,” “fire,” “girl,” “ladder,” and “stuck” (see Table 6). For all items, individuals with moderate aphasia demonstrated a longer median production time than HCP. For two items (“climb” and “department”) survival curve comparisons could not be completed because fewer than 10% of individuals with moderate aphasia produced the CoreLex item.

##### 4.4.1.4 Severe aphasia

Only two individuals with severe aphasia produced a CoreLex item in the top right quadrant, so they were excluded from the analysis in that quadrant but were included in the others. In the *top left* quadrant, the first CoreLex item was produced by half of individuals with severe aphasia by 21.5 s (χ^2^ = 38.265, *p* < 0.001). In the *bottom left* quadrant, the first CoreLex item was produced by half of individuals with severe aphasia by 22.9 s (χ^2^ = 5.02, *p* = 0.025). In the *bottom right* quadrant, the first CoreLex item was produced by half of individuals with severe aphasia by 27.735 s (χ^2^ = 6.319, *p* = 0.012). Survival curve comparisons could only be completed for the items “cat,” “dog,” and “father” because fewer than 10% of individuals with severe aphasia produced the other CoreLex items. When comparing the survival curves of HCP and individuals with severe aphasia for these items, significant differences were seen for all three (see Table 6). For all items, individuals with severe aphasia demonstrated a longer median production time than HCP.

##### 4.4.1.5 Profound aphasia

Individuals with profound aphasia had limited output: only 2 of these individuals produced a CoreLex item in the top left quadrant, none produced an item in the top right, and only one produced an item in the bottom left and bottom right quadrants. Individuals with profound aphasia were not included in these analyses since the small sample size was not appropriate for statistical treatment.

#### 4.4.2 Order of core lexicon production

Despite the consistent order of CoreLex production when comparing HCP to all PWA, the data reveal a different story when PWA are separated into groups by severity (see the third through seventh columns in Figure 4). Individuals with latent aphasia produced the first two CoreLex items in the same order as HCP, but all other items were offset from the HCP order by one position (except “stuck” which individuals with latent aphasia produced two items before HCP). Individuals with moderate, severe, and profound aphasia also produced most CoreLex items in a different order from HCP. Individuals with moderate and profound aphasia only produced the first CoreLex item in the same order as HCP. Individuals with severe aphasia produced two items in the same order as HCP. However, there were no clear patterns in the differences between the aphasia groups and HCP. Sometimes two CoreLex items were simply reversed by individuals with aphasia, but other items were produced in a very different order. For example, individuals with moderate aphasia who produced “department” said it as the third CoreLex item in their stories, while HCP produced it as the second to last item. In contrast to the other groups, individuals with mild aphasia more closely matched HCP’s order, producing all but four items in the same order. Individuals with mild aphasia reversed the order of “climb” and “father, “department,” and “bark” compared to HCP.

## 5 Discussion

The current study was anchored in the premise that analyzing discourse production performance during a narrative picture description task may offer a promising avenue to understand the nature of visuospatial processing and language production in PWA. When comparing CoreLex scores across groups, our results were consistent with previous research which has identified significant differences between HCP and PWA, as well as between HCP and groups of individuals with different types or severities of aphasia. This provides assurance that additional analyses of the data are appropriate.

### 5.1 Visuospatial processing and CoreLex production

The close alignment in the order of attending to the quadrants for first CoreLex word production points to the notion that PWA are processing the visual scene elements in a similar manner compared to HCP, nonetheless with a general slowing in either attending to or producing the most salient CoreLex items within each quadrant. Given that CoreLex words reflect important lexical items required to construct a meaningful and coherent narrative, a consistent pattern of delay in their production is indicative of the lexical retrieval challenges experienced by PWA at the discourse level (Kim and Wright, 2020). Aim 1 results are also in line with findings from Dalton and Richardson (2015), who have posited that difficulties in lexical retrieval potentially contribute to a reduction in both the quality and quantity of discourse performance, as PWA are less likely to construct complete, informative, and cohesive narratives in the face of ongoing difficulties in accessing and producing highly relevant vocabulary (e.g., CoreLex). They are also less likely to attempt producing lexical items that they believe to be erroneous. These lexical retrieval challenges are further compounded by two important factors, which may negatively influence CoreLex production. First, given that a discourse task subsumes contextual factors, cognitive abilities necessary for attending to and processing context may create additional “knock-on” lexical retrieval difficulties beyond those directly attributed to aphasia (Kim et al., 2019). Second, lexical retrieval challenges in aphasia can overstrain the working memory (WM) system, as the time interval between narrative conception and production continues to increase without production of CoreLex words, which imposes additional cognitive load, leading to an impaired production of already retrieved words appearing in the later part of the narrative (Dalton and Richardson, 2015). This notion is further strengthened by evidence suggesting that PWA are significantly disadvantaged in maintaining visuospatial WM for object identity and location, when engaged in a verbal processing task, possibly due to left hemisphere damage, which may disrupt both language processing and nonlinguistic cognitive functions, including visuospatial processing (Cohen-Dallal et al., 2022; Hillis, 2007; Smith and Jonides, 1999).

This relationship between visuospatial skills (especially visuospatial WM) and language production in PWA may also have potential clinical utility in aphasia management including assessment and intervention. There is evidence to suggest that PWA completing a speech-language focused aphasia rehabilitation program of duration as short as 3 weeks have shown significant improvements in language functions, accompanied by improvements in visuospatial WM, further highlighting the positive correlations of visuospatial WM with language intervention outcomes (Diedrichs et al., 2022; Seniów et al., 2009). Production of the first CoreLex item in each quadrant may also be a function of participants using specific spatial strategies. In sentence comprehension studies of aphasia, it is documented that when English-speaking PWA are engaged in sentence-picture matching or verification tasks, they tend to use spatial strategies which prefer attending to stimuli in the left visual field before moving on to items in the right visual field, especially when event pictures are used (Chatterjee et al., 1995; Chatterjee et al., 1999; Mack and Thompson, 2017; Mitchum et al., 2010). Results of the current study are in line with this evidence, further highlighting the deeper connections between visuospatial abilities and discourse performance, as well as the role of CoreLex analysis as a clinical assessment tool in indexing this relationship in an objective manner.

### 5.2 Timing of CoreLex production

Except for “fireman,” PWA required significantly more time to produce CoreLex items than HCP. While it is possible that group differences in the time taken to produce a CoreLex item are related to demographic or psycholinguistic variables such as word frequency, it is unlikely that these factors fully account for the observed results. With respect to demographic variables, while age and education are associated with word-finding abilities, our results demonstrated that even after controlling age and education, group differences persisted, indicating that basic demographic factors alone do not fully account for the differences. Basic psycholinguistic variables, such as word frequency and word length, also do not appear to account for these results. For instance, “cat” and “girl” are both high frequency words. While “cat” showed one of the shortest delays (~8 s) between the PWA and HCP, “girl” had one of the longest delays between groups (~19 s). Similarly, the difference in median time to production for longest CoreLex word, “department,” was ~14 s, in contrast, the median time to production for one of the highest frequencies (and phonologically simplest) CoreLex items, “dog” was ~19 s.

Additionally, group differences in time to production do not appear to be directly related to the order of production of CoreLex items. For example, at least 50% of HCP and PWA produced “girl” as the second CoreLex item, and the delay between groups was ~19 s, while at least 50% of both groups produced “fireman” last, and the delay between groups was ~10 s. These results also suggest that time to production is not explained by a delay in task onset by PWA compared to HCP (where we would expect the difference between groups to be the same or similar for all CoreLex items) or a slower speaking rate for PWA compared to HCP (where we would expect a gradually increasing difference in the time to produce each item between groups). Rather, it seems that cognitive processes, whether language specific or more general, are driving significant differences in production time across groups. These findings demonstrate that understanding the timing of production of CoreLex items may improve sensitivity to detect differences, which is in line with research demonstrating that discourse efficiency may be more sensitive than count or frequency discourse measures (e.g., CIU/min, CoreLex/min; Nicholas and Brookshire, 1993; Dalton et al., 2020a).

### 5.3 Aphasia severity effects timing and order of CoreLex production

The delay in CoreLex word production between HCP and PWA varied according to severity, wherein PWA with greater severity typically produced CoreLex items at a greater delay. This finding was expected based on results from previous CoreLex discourse studies involving picture description scenarios in aphasia (Dalton and Richardson, 2015; Dalton et al., 2020a; Kim et al., 2019; Kim et al., 2021; Kong and Wong, 2018) as well as those with MCI and Probable AD (Fromm et al., 2024). In terms of the order of attending to visual stimuli across the quadrants, we observed that across all severity groups PWA showed an order of CoreLex word production that was similar to HCP: a counter-clockwise pattern in the order of *top left → bottom left → bottom right → top right.* This finding supports the notion that PWA may still retain the overall strategic ability to plan, organize, and produce discourse at a higher conceptual level despite ongoing language impairments (Dalton and Richardson, 2015). It also reflects the dissociation between cognitive planning stages of discourse, and the stage of language production. That is, PWA may have spared abilities of identifying *what* to describe and broadly in what *order*, despite difficulties in the accuracy and speed of language production. Finally, results may have potential implications for developing targeted interventions in aphasia management, wherein the goals and structure can focus on improving efficiency of lexical access and sentence formulation, relying on spared higher-level organizational structure (Diedrichs et al., 2022; Kim and Wright, 2020).

While individuals with severe aphasia showed a significant delay in producing the first CoreLex item in the top left quadrant, once the first CoreLex item in the top left quadrant had been produced, they very quickly produced an item from the bottom left and bottom right quadrants. However, almost none produced a CoreLex item from the top right quadrant. There is no previous research documenting this pattern of potential facilitation of subsequent CoreLex word production following challenges producing the first CoreLex word in picture description contexts. Additionally, individuals with latent aphasia produced CoreLex items in each quadrant at a significant delay compared to HCP (Figure 5). These results are in alignment with previous research highlighting the importance of CoreLex analysis as a sensitive tool in capturing language production in people with latent aphasia, who might otherwise show comparable performance to HCP in traditional language productions tasks (Dalton and Richardson, 2015). Our findings, together with previous research investigating latent aphasia represent a growing repertoire of valid measures for indexing language problems in people with latent aphasia. For example, people with latent aphasia show increased pauses in spoken discourse production (Salis and DeDe, 2022), produce more word errors, reduced lexical diversity, fewer utterances (Fromm et al., 2017), and reduced informativeness (Dalton et al., 2020a; Fromm et al., 2017).

A notable finding of the differences in aphasia severity was related to the order of CoreLex production. When comparing HCP to the entire group of PWA, CoreLex items were mostly produced in the same order. This similarity would support broadly intact visuospatial processing in the entire group of PWA. However, the individual severity groups produced CoreLex items in more variable orders, apart from individuals with mild aphasia. Given that individuals with latent aphasia were as likely as individuals with moderate and severe aphasia to produce CoreLex items in a different order than HCP, it seems unlikely that differences are directly related to overall aphasia severity. Nor does a comparison of CoreLex production order with quadrant locations seem to explain the observed differences across aphasia severity groups. For example, no group produced all the items in one quadrant, then all the items in another quadrant, etc. Rather, items from different quadrants were intermixed with one another, better reflecting the flow of the story shown in the picture. Additional research could elucidate the factors driving these order differences.

### 5.4 Study limitations

While we can draw some inferences between visuospatial behavior and timing of spoken language production, our interpretation of these results is restricted without direct measurement of eye movement behaviors when viewing the stimulus of interest. It is difficult to determine how an individual’s eye movements during stimulus viewing influences their subsequent discourse performance. For example, reading comprehension and eye-tracking studies in PWA suggest that repeated fixations on previously attended words indicate word processing difficulties (Knollman-Porter et al., 2023) or a memory support or confirmation strategy (Hux et al., 2024). In discourse tasks, especially those engaging visuospatial behaviors, such as picture description tasks, it is not known what refixations to specific areas of interest mean during speech production (e.g., difficulties in lexical access as opposed to CoreLex production, or both). In the absence of eye-tracking measures we can only hypothesize about underlying mechanisms based on previous literature. However, findings from the CoreLex analyses point to the notion that visuospatial processing may be more closely interrelated to discourse performance than previously documented (Diedrichs et al., 2022; Lee et al., 2020).

Next, the decision to divide the picture scene into four quadrants for analysis was made *a priori*. However, it is possible that participants process the image in a different fashion. Eye-tracking measures would elucidate whether a quadrant-based approach or another alternative is more appropriate. The use of a single picture description task provides limited information regarding CoreLex word production in PWA. Given the inherent differences between discourse performance when using single pictures (which elicit greater descriptive statements) as opposed to picture sequences (which elicit greater narrative statements), it will be important to include multiple types of picture description tasks when assessing discourse performance in PWA using pictures (Dalton and Richardson, 2015; Olness et al., 2002). For example, including a combination of a single picture scene description (e.g., Cat Rescue), as well as a four-picture sequence description (e.g., Broken Window) will provide richer and more complete discourse performance data for PWA (Dalton et al., 2024).

Although significant efforts are underway to increase the diversity of language samples in AphasiaBank, the current sample still predominantly consists of Caucasian, monolingual English speakers. This limitation restricts the generalizability of the findings to other populations. On a related note, exclusion of synonyms in the CoreLex analysis discounts semantically related alternative productions to CoreLex items or lexical substitutions, potentially underrepresenting lexical diversity, and communicative competence of PWA. This constraint may disproportionately affect individuals from diverse linguistic or cultural backgrounds, whose lexical choices may naturally vary from the CoreLex targets. Finally, the designation of a core lexicon item was based on previously published literature, which reported a core lexicon checklist based on the production of a large sample of HCP (Dalton et al., 2020a,b). We used the items reported in this checklist, regardless of the proportion of participants in our groups who produced the items. In the current study, there were two items produced by fewer than 50% of the HCP group: “bark” which was produced by 46.4% of HCP and 39.3% of PWA, and “fireman” which was produced by 38% of HCP and 44.3% of PWA. Continued expansion of the discourse samples from HCP and PWA will be important in addressing these issues and identifying a homogenous sample of CoreLex words for future research.

### 5.5 Future recommendations

Future research exploring the connections between visuospatial processing and discourse performance will be important for obtaining crucial insights into the nature of visuospatial and cognitive processing abilities of PWA across all severity levels, including the mildest of aphasias and developing a more holistic view of communication abilities and limitations of PWA, going beyond just linguistic measures. There are several avenues for integrating visuospatial tasks into discourse-based assessments and treatments in PWA. When using single picture description tasks (e.g., Cat Rescue), clinicians can evaluate how PWA perceive and interpret visual scenes by noting any difficulties beyond linguistic analysis (e.g., CoreLex production). These include challenges with figure-ground analysis (differentiating main visual elements from the background), agnosia (recognizing objects or people in the picture), or visual perceptual organization, including the understanding of spatial relationships and overall context of the scene (Fromm et al., 2024). It has been speculated that difficulties with lexical retrieval in PWA when engaged in picture description tasks may place strain on working memory capacity, negatively influencing the overall discourse performance (Dalton and Richardson, 2015). Visuospatial behaviors, including those of refixations to previously attended items may be influenced by a common underlying process. Therefore, future studies in this line of research should also integrate working memory and attentional measures to obtain more clarity on the cognitive underpinning of discourse performance. On a related note, clinicians can also leverage spared visuospatial abilities in PWA to identify and build visual stimuli strategically into the picture, to support discourse abilities by reducing the cognitive load required to process specific visual elements required to produce discourse. For instance, placing picture elements within a scene in a strategic way to promote understanding of spatial and temporal relationships while narrating a story. Treatment methods combining picture description tasks can be used to develop novel and individualized therapeutic interventions, for improving CoreLex word production, through serial presentation of different crucial lexical items within the scene that unfold in due course of time.

Additionally, this line of research has potential to facilitate expansion of current discourse-based tools for aphasia management. Eye-tracking evidence from syntactic processing studies suggests that PWA show improved lexical access when provided with additional time when processing crucial lexical items in auditory comprehension tasks (Baker and Love, 2021, 2023). There is limited evidence regarding whether such facilitation to lexical access occurs in speech production contexts. Discourse assessment tasks integrating eye movement measurements offer a promising paradigm to investigate the nature of lexical access during speech production, as discourse production is not subject to external temporal constraints (unlike structured comprehension tasks) and participants typically complete discourse tasks in a self-paced manner. These tasks may also be beneficial in tailoring aphasia treatment dosage to suit individual goals. Finally, it will be important to develop experimental paradigms that can accommodate picture description tasks that are personally meaningful for PWA (e.g., personal photographs), which can stimulate personal narratives and are also reflective of everyday communicative contexts (Dalton and Richardson, 2015).

## 6 Conclusion

The current study offers key insights into the nature of underlying connections between visuospatial skills and language performance in PWA, emphasizing the crucial role of cognitive abilities in language production in aphasia than previously attributed, as well as the similar manner PWA process visual scene elements compared to HCP despite delays in CoreLex word production. The results offer preliminary evidence for clinical utility of discourse tools in measuring visuospatial behavior during language performance in PWA. Specifically, the results highlight the CoreLex analysis as an important clinical tool for concurrent assessment of visuospatial processing in addition to aphasic discourse. Given the corpus of previous evidence demonstrating the deeper connection between visuospatial skills and language performance in PWA, it will be important to further expand and refine discourse-based assessment by incorporating visuospatial assessment tools, such as eye-tracking methods in improving rehabilitation outcomes for PWA.

## Data Availability

Publicly available datasets were analyzed in this study. This data can be found here: the data reported here are available to members of the AphasiaBank consortium (https://aphasia.talkbank.org/). Established researchers and clinicians working with aphasia who are interested in joining the consortium should read the Ground Rules (https://talkbank.org/share/) and then send an email to macw@cmu.edu with contact information, affiliation, and a brief statement explaining reasons for joining.
